# *Escherichia coli*: a brief review of diarrheagenic pathotypes and their role in diarrheal diseases in Iran

**Published:** 2012-09

**Authors:** A Jafari, MM Aslani, S Bouzari

**Affiliations:** 1 Molecular Biology Unit, Pasteur Institute of Iran, Tehran; 2 Bacteriology Department, Pasteur Institute of Iran, Tehran; 3 National Escherichia coli Reference Laboratory (NERL)

## Abstract

Diarrheagenic *Escherichia coli* have developed different strategies for establishment of infection in their host. Understanding these pathogenic mechanisms has led to the development of specific diagnostic tools for identification and categorization of *E. coli* strains into different pathotypes. This review aims to provide an overview of the various categories of diarrheagenic *Escherichia coli* and the data obtained in Iran pertaining to these pathotypes.

## INTRODUCTION

Despite the fact that *Escherichia coli* as a commensal bacteria can be found in intestinal microflora of a variety of animals including man, not all the strains are harmless, and some can cause debilitating and sometimes fatal diseases in humans as well as mammals and birds (1). Pathogenic strains are divided into intestinal pathogens causing diarrhea and extraintestinal *E. coli* (ExPEC) causing a variety of infections in both humans and animals including urinary tract infections (UTI), meningitis and septicemia (2).

Cystitis and pylonephritis that can lead to urosepsis is caused by Uropathogenic *E. coli* (UPEC) which are the cause of approximately 80% of the estimated 130-175 million human UTIs (3). Furthermore, ExPEC are the primary gram-negative bacterial pathogens associated with neonatal meningitis and are the second overall cause of the disease after group B Streptococci (4, 5). Severe neurological lesions resulting from infection with meningitis-associated *E. coli* (MNEC) leads to death in 20-40% of infected infants (5). Nosocomial bloodstream infections in hospitals and nursing homes may be caused by ExPEC strains which may also be the cause of respiratory, UTI or bacterimia in long-term hospitalized patients (6). Resistance to antimicrobials has made combating these infections a major problem worldwide (7).

The main focus of this review however is diarrheagenic *E. coli* and therefore ExPEC will not be further discussed.

An altered movement of ions and water following an osmotic gradient is at the heart of diarrheal diseases. Under normal conditions, the capacity of gastrointestinal tract to absorb fluid and electrolytes is tremendous and from 8-9 liters of fluid presented to intestine daily, only 100-200 ml are excreted in the stool. Enteric pathogens, however, can alter this balance towards net secretion leading to diarrheal disease (8). Diarrheagenic *E. coli* (DEC) strains are among the most common etiologic agents of diarrhea and based on their specific virulence factors and phenotypic traits are divided into enteropathogenic *E. coli* (EPEC), enterotoxigenic *E. coli* (ETEC), Vero toxin-producing/Shiga toxin-producing *E. coli* (VTEC/STEC) which include its well-known subgroup enterohaemorrhagic *E. coli* (EHEC), enteroinvasive *E. coli* (EIEC), enteroaggregative *E. coli* (EAEC), and diffusely adherent *E. coli* (DAEC). In this review, we have attempted to summarize recent findings concerning different pathotypes of diarrheagenic *E. coli* including data published from Iran as they continue to be important causes of disease in both the developed and developing world. Furthermore presentation of the information from Iran along with the main body of results gathered elsewhere may help to identify the gaps in our knowledge related to these pathogens. Articles cited from Iran in this review are limited to those that were in English and accessible through PubMed or ISI sites and no other exclusion criteria were implemented.

### Enteropathogenic *Escherichia coli* (EPEC)

#### History

Until the 1970s serotyping was the only means of distinguishing EPEC strains from those of normal flora, since no biochemical, microbiological or animal tests were available for their differentiation (9). The 12 serogroups originally recognized by the World Health Organization as EPEC or the classical EPEC were; O26, O55, O86, O111, O114, O119, O125, O126, O127, O128, O142 and O158 (10). Current classification of EPEC however, is based on the presence of specific virulence genes, which the use of molecular techniques has shown to be present in serogroup/serotypes other than classical ones as well (11).

#### Pathogenesis

The distinctive histopathology induced by this group of *E. coli* is termed attaching and effacing (A/E) lesions and is caused by the intimate attachment of bacteria to the intestinal epithelial cells and effacement of enterocyte microvilli (12). Formation of the micro ulcers and exfoliation of the cells at the site of EPEC attachment was first described in experimentally infected pigs (13) and subsequently in biopsies from infected infants (12). A protein called intimin mediates the bacterial attachment to outer cell membranes and is encoded by *eae* gene which along with all other genetic elements required for this phenomenon are located on the locus of enterocyte effacement (LEE), a large genomic pathogenicity island which was discovered in 1995 (14). The *eae* is one of the genes currently used for the molecular diagnosis of EPEC.

Pathogenesis of these bacteria however is many faceted which has not been fully unraveled as yet and may involve factors other than those directly responsible for A/E lesions as well as more specialized intestinal cells (15, 16).

#### Detection

Originally, HEp-2 cell-adherence assay performed with serologically defined EPEC strains showed that 80% of these strains adhere to HEp-2 cells in vitro (15). The HEp-2 assay has been modified often since its first description, including such variations as extending the incubation time to 6 h or changing the growth medium during the incubation. However, collaborative studies have shown that the assay performed essentially as first described provides the best ability to differentiate among EPEC, EAEC, and DAEC isolates (16).

After the introduction of the term “attaching and effacing” actin accumulation under the attached bacteria was demonstrated using *ex vivo* culturing of human intestinal biopsies (17). Staining this electron dense material produced the actin fluorescent assay (FAS) which enabled researchers to detect the ability of a strain to produce A/E lesions in vitro (18). It should however be noted that a negative FAS result may depend on the cell type used and the bacteria should be confirmed as nonpathogenic by alternative methods (10).

The localized adherence pattern of EPEC strains was shown to be associated with the presence of a 60 MDa plasmid called pMAR2 from which a DNA fragment of 1 kb was isolated which has been used extensively in epidemiological studies (19–21).

The presence of the *E*. *coli* adherence factor (EAF) plasmid carrying *bfp* operon, encoding the type IV bundle-forming pilus (BFP), and *per* operons, a transcriptional activator called plasmid encoded regulator (Per) is the basis of typical and atypical classification of EPEC strains (22). All EPEC strains lack genes encoding Shiga toxin (*stx*) although they share A/E phenotype with some other strains of *E. coli*, therefore, strains that are *eae*+ *bfpA*+ *stx*- are classified as typical EPEC (tEPEC). Production of BFP protein induces the localized adherence pattern (LA) and most of tEPEC strains belong to classic O:H serotypes (22). Atypical EPEC (aEPEC), on the other hand, are of *eae*+ *bfpA*- *stx*- genetic background and display localized-like (LLA), diffuse (DA), or aggregative adherence patterns which is associated with the *E. coli* common pilus and other known adhesins (23). Most of the over 200 O-serogroups that have been identified among aEPEC strains, do not belong to classical EPEC serogroups and many have been designated nontypeable (24). Recently in a study done in Iran, multiplex PCR was used to differentiate between tEPEC, and aEPEC and PCR-RFLP for H typing of conventionally serogrouped isolates (25) showing the ease and applicability of this method for rapid screening of large number of isolates.

#### Epidemiology

Although EPEC are among the most important pathogens infecting children less than 2 years of age in the developing world, but the prevalence may vary depending on differences in study population, age group, diagnostic criteria and diagnostic tools used (11). Over the last several decades, the significance of EPEC infection has declined in published literature. The decline might be due to interventions, particularly breast-feeding promotion, or to the overestimation of these organisms in earlier studies that used O- or O:H typing compared to the recent ones, in which molecular methods and/or adherence assays was used for EPEC diagnosis (26–27). A study conducted in the south of Iran (28) using serological test alone for identification of EPEC reported an isolation rate of 30.7% for this pathotypes in children of less than 3 years old with diarrhea. The isolates belonged to 12 different serogroups, of which O128 and O126 were the most prevalent. A similar study published 2 years later reported EPEC as the most common pathogen among patients admitted to 4 children's hospital in Tehran and those attending an outpatient clinic in Sanandaj with an isolation rate of 26.7 and 20.1% respectively (29). Recent studies dealing with diarrhea in children under 5 years of age have reported varying rates of isolation for EPEC ranging from 12.6-44.9% showing a significant association between EPEC isolation and diarrhea (30–33). A noticeable reduction in isolation rate of EPEC was reported in 2005 in which 200 children suffering from acute diarrhea with age-matched controls were studied in Tehran and EPEC was isolated from 6% of patients and 5% of controls (34). The reason for the low prevalence of EPEC in this study was not discussed.

Various studies carried out in Iran and abroad have shown a significant association between EPEC and infant diarrhea compared to controls (30–33, 35–36), but similar isolation rates from diarrheal cases and controls have also been documented (34). However, in none of these studies the typical and atypical EPEC have been separately addressed, except for the work by Alikhani *et al*. (30). The data obtained showed a significant association between both typical and atypical EPEC and diarrhea. In both developed and developing countries an increasing isolation rate for aEPEC has recently been reported, but the epidemiological association of this group with childhood diarrhea is controversial and requires further studies (27).

In developing countries healthy carriage of enteric pathogen is shown to be common and has been attributed to the interplay of many factors including host susceptibility (related to the child's age, breastfeeding, nutritional and immunological status), bacterial virulence factors (different virulence genes), and environmental factors (poor hygiene, and high fecal contamination). However, data from a recently developed quantitative real-time PCR (qRT-PCR) has shown a significantly higher bacterial load in EPEC-associated diarrheal samples compared to controls (27).

Prolonged and persistent episodes of diarrheal disease (diarrhea > 14 days) constitute a significant portion of the global burden of diseases in children (27). A recent systematic review of the illness in developing countries has shown that EPEC, ETEC and EAEC are the main pathogens associated with this complication, and are responsible for 30-40% of all persistent diarrheal episodes in children (37). In developed countries, aEPEC is the most common pathogen isolated from children with persistent diarrhea accounting for more than half of the episodes (38). The role of aEPEC in persistent diarrhea in Iran has not been dealt with systematically.

### Enterotoxigenic *E. coli* (ETEC)

#### History

ETEC is the most important but under-recognized bacterial cause of diarrhea or cholera-like disease in all age groups in areas with poor sanitation and inadequate clean water. Furthermore, of the estimated one billion yearly international travelers, 20-60% of those traveling to low-income countries will suffer from travelers’ diarrhea (39). In approximately 30-70% of traveler's diarrhea bacteria are the causative agent, of which ETEC are the most commonly detected (40). Development of rabbit ileal loop assay which led to the discovery of cholera toxin was also used for pure cultures of *E. coli* isolated from stools and small bowels of children and adults showing similar symptoms to cholera. Live cultures and culture filtrates of these strains when injected into isolated rabbit ileal loops produced strong cholera-like secretary response leading to the discovery of the heat-labile enterotoxin of *E. coli* and recognition of ETEC pathotype in 1968 (41–42).

#### Pathogenesis

ETEC strains adhere to intestinal epithelial cells via a heterogenous group of proteinacious surface structures termed colonization factors (CFs) which can be fimbrial, non-fimbrial or fibrillar (43). The more recent nomenclature refer to these structures as coli surface (CS) antigen, but some of the old names still persist such as colonization factor antigen I (CFA/I). Despite the fact that more than 25 CFs have so far been identified, on many strains no CF is detected which might be attributed to the technique (s) used for detection, true lack of CFs or as yet unidentified ones (44).

Following the initial adhesion and colonization, ETEC strains cause diarrhea by producing heat-labile (LT) and/or heat-stable (ST) enterotoxins, which are plasmid-encoded (45). ETEC bacteria produces the small STs as a 72-amino acids preprotoxin which is processed into an 18-19 amino acid active toxin called STa and a 42 amino acid toxin referred to as STb. STa is produced by both human and animal strains, whereas STb is mainly detected in strains of veterinary origins (46). LT like the closely related cholera toxin is a member of AB_5_ family of toxins which are heterohexameric molecules consisting of five B subunits and a single A subunit (47). The A1 domain constitutes the active toxin and is linked to the A2 domain via a disulfide bond (48). The A2 fragment is the helical portion of the molecule and anchors the A subunit to the B pentamer which binds irreversibly to GM1 ganglioside as receptors on cell surface (46). The toxin is then internalized and the A subunit ADP-ribosylates the stimulatory guanine-nucleotide-binding protein, increasing the levels of intracellular cyclic AMP resulting in diarrhea.

#### Detection

Diagnosis of ETEC is based on the production of LT and/or ST and the rabbit ileal loop and infant mouse physiological assays were initially used as gold standards for the identification of these enterotoxins respectively. These tests are difficult to perform and time consuming and for a while efforts were made to use serotyping for this purpose, but soon it became clear that a large number of serotypes could be enterotoxigenic and therefore not applicable (49). In 1974 it was found that LT produces morphological changes on Y1 adrenal and Chinese hamster ovarian cell lines that were neutralizable by antitoxin (50–51). Although these tissue culture tests were used in preference to the animal models, but these assays were only useful for LT detection and not available in all laboratories making ETEC detection problematic. Enzyme-linked immunosorbant assay, passive latex agglutination, immunoprecipitation in agar and Biken test were developed subsequently and were found to be specific (52–54). PCR has revolutionized clinical diagnosis of pathogenic microorganisms and was used in 1994 for detection of ETEC strains (55) but prior to the advent of PCR methods radioactively and nonradioactively labeled probes were used for detection of enterotoxin genes and the method was shown to be both sensitive and specific (49, 56–57).

Different methods have been used for CF detection, including mannose-resistant agglutination of certain species of erythrocytes, serological tests initially using polyclonal sera (58–60) which were subsequently replaced by monoclonal antibodies and eventually molecular methods (61). These methods were used in a study conducted in Iran to characterize ETEC strains with ten years difference in isolation date and a high degree of agreement was observed between the results obtained using different detection methods (62). In 1992 however, both mannose-resistant hemagglutination and polyclonal antisera for CFA/I and CFA/II were used for detection of these antigens among Iranian ETEC isolates and it was concluded that hemagglutination was not specific enough for characterization of these fimbriae (63).

Besides determination of the toxins and CFs, serotyping, i.e. determination of O serogroups associated with the cell wall lipopolysaccharides and H serogroups of the flagella, has been applied for identification and characterization of ETEC (64). However, as shown in studies conducted in different countries, clinical ETEC isolates may belong to a large number of serotypes making this method unsuitable for identification of these bacteria. Furthermore, ETEC serotype profiles may change over time (49).

#### Epidemiology

The first report of ETEC in a case series of infantile diarrhea in Iran was made in 1982 when Mohadjer *et al*. (65) detected LT and ST in the isolates using rabbit ileal loop, Y1 adrenal cell culture and infant mouse assay. The isolation rate for this pathogen was 7% and subsequently using rabbit ileal loop and infant mouse assay in a case series study ETEC was detected in 21.9% of diarrheal cases attending 12 outpatient clinics in Bandar Abbas (28). Detection of various toxin genes among 200 *E. coli* isolates from diarrheal cases in Tehran using Dig-labeled probes showed that LT and ST carrying isolates were the least frequent pathotypes (66). Recently, however, PCR has been the detection technique of choice and the rate of ETEC in diarrheal cases in studies using this method varied from nearly 33% (67) and 15.5% (34) to less than 10% (32, 33, 68).

Unfortunately, despite the availability of various techniques, there are still no simple, readily available methods for identification of these organisms in minimally equipped laboratories or the field. Therefore, ETEC is not included in routine diagnosis of diarrhea in many laboratories.

### Shiga toxin-producing/ Enterohaemorrhagic *E. coli* (STEC/EHEC)

#### History

The main virulence factor and the defining feature of this group is a phage-encoded potent cytotoxin the effect of which was shown to be neutralizable by anti-Shiga toxin of *Shigella dysenteriae* 1. The cell toxicity effect was also demonstrated on Vero cells resulting in a parallel nomenclature system of Shiga/Vero toxin-producing *E.coli* (STEC) and (VTEC) respectively (16, 69).

In 1983, an *E. coli* strain serotype O157:H7, was identified in association with outbreaks of a bloody diarrhea called hemorrhagic colitis (HC) leading to the recognition of EHEC as a new and increasingly important class of enteric pathogens causing intestinal and renal disease (16). The term enterohaemorrhagic *E. coli* (EHEC) is applied to those STEC serotypes that have the same clinical, epidemiological and pathogenetic features associated with the prototype strain *E. coli* O157:H7.

The high virulence of STEC strains such as O157:H7 is not only dependent on the virulence factors but partially also on the pathogen's ability to survive environmental stress conditions, such as resistance to low pH levels found in the gastrointestinal tract contributing to its very low infectious dose of 50-100 bacteria or lower (70).

Among STEC serotypes, O157:H7 is associated with both outbreaks and sporadic cases of severe disease, but it has been shown that other serotypes may also cause human infections albeit variably (71). This quantitative and qualitative difference in disease association among STEC has given rise to various classification schemes the simplest of which divides STEC into *E. coli* O157 and non-O157. However, in view of the fact that the virulence potential of non-O157 might be genetically determined a seropathotype (SPT) classification has been proposed in which prior association with human epidemics, HUS, and diarrhea is considered (71). In this scheme, SPT-A includes O157:H7 and O157:NM, the most commonly isolated serotypes from outbreaks and HUS. SPT-B strains differ from group A in the frequency of isolation from outbreaks and HUS cases, SPT-C strains are only associated with sporadic cases of HUS, SPT-D are isolated from diarrheal cases and have not been encountered in outbreaks or HUS and SPT-E that have never been associated with human disease (72). Furthermore, data collected using different methods of comparative genomic have suggested that several discreet genotypes differing in virulence exist within *E. coli* O157:H7 population and based on these data this serotype has been subdivided into nine clades (73–74).

Recently a new Shiga toxin producing *E. coli* strain was identified in Germany causing one of the largest outbreaks of HUS worldwide. The perpetrator belonged to serotype 104:H4 which contained the virulence factors of typical EAEC and a Stx-2 producing prophage, but lacked the LEE pathogenicity island. This discovery has led to the emergence of a new pathotype for which the name Entero-Aggregative-Haemorrhagic *Escherichia coli* (EAHEC) has been suggested (75–76) This event exemplifying the genome plasticity of *E. coli* has highlighted the need for public health surveillance of STEC infections and its important role in devising and implementing control measures.

#### Pathogenesis

Shiga toxin family with related structure and similar biological activity is composed of Stx1 which is essentially identical to the toxin of *Shigella dysenteriae* differing in a single amino acid and Stx2 with less than 60% amino acid homology to Stx1 (47, 77). Little sequence variation has been reported for Stx1 (78), but Stx2 has several subtypes which differ in biological activity and immunological reactivity (79). Shiga toxins similar to the heat-labile enterotoxin of ETEC belong to the AB_5_ family of the toxins and consist of a pentameric ring-shaped B subunit that is non-covalently attached to the A subunit. The B subunit interacts with globotriaosylceramides (Gb3s) on the surface of human intestinal mucosa and kidney epithelial cells resulting in the internalization of the toxin where the A subunit is activated causing cell death (43). Among the Stx2 variants, Stx2c has been isolated more frequently from HUS patients but Stx2e and Stx2f have been mainly isolated from pigs and birds and rarely from humans (77). Moreover, a different AB_5_ toxin has been discovered in this group which differs significantly from other toxins in this group. This subtilase-like toxin (SubAB) was isolated from an HUS outbreak strain in Australia and shows greater cytotoxicity than Stx2 for a range of cell types including Vero cells (47).

The EHEC genome contains the same locus of enterocyte effacement (LEE) as the EPECs and the intimate attachment of EHEC to host cells occur through interaction between an adhesin called intimin (*eaeA*), and Tir (translocated intimin receptor). This intimate attachment induces the characteristic attaching and effacing lesions (A/E), but the initial adherence of EHEC to colonocytes is not well defined (43, 45, 70, 77). Sixteen potential fimbria-like operons, which have not been extensively studied have been recognized in STEC strains (43, 80), and recently a pilus involved in adherence and biofilm formation called hemorrhagic coli pilus, a type IV pilus, has also been identified in STECs (81). However, the intimate adherence as in EPECs is through the interactions between Tir and intimin. At least 29 distinct intimin types with heterogeneity in the C-terminal part of the molecule that is involved in binding to Tir in both STEC and EPEC have so far been identified (82–83). The ability of STEC to produce A/E lesions is sufficient to cause non-bloody diarrhea but Shiga toxin is essential for the development of bloody diarrhea, HC, and HUC (16, 84). Another toxin found in many STEC/EHEC isolates is the enteroaggregative heat-stable enterotoxin1 (EAST1) and usually two copies of the *astA* gene is present in the chromosome (16, 70). The significance of this carriage in the pathogenesis of EHEC is unclear, but it has been suggested that some of the non-bloody diarrhea in person infected with these strains might be due to the production of this toxin (16, 83). The primary virulence determinants of EHEC strains are chromosomally encoded, but plasmids might play an important role in the pathogenesis of EHEC strains. Plasmid pO157 is found in 99-100% of O157:H7 serotype isolated from human clinical isolates, most not all Stx-producing isolates. Presence of this plasmid has been correlated with hemolytic activity and adherence to intestinal epithelial cells, but the overall understanding of the role of plasmids in pathogenesis of STEC/EHEC strains is hindered due to the absence of a reliable model of human infection (45, 85–86).

#### Detection

Laboratory confirmation of STEC infection can be achieved by isolation and confirmatory tests using culture media, immunoassays, cell toxicity assays and PCR (87–88). Screening of O157 relies on the strain's inability to utilize sorbitol rapidly, leading to the use of sorbitol-MacConkey agar (SMAC) as a differential medium with added cefixime and tellurite (CT-SMAC) although in our setting addition of cefixime has not led to the prevention of other fecal-associated microorganisms. More specific media have also been developed such as Rainbow agar, CHROMagar^®^, and O157:H ID agar that are able to recover O157 along with sorbitol-fermenting O157 and non-O157 strains (70, 88). Tests conducted in our laboratory, however, has shown that to prevent the growth of some bacterial strains such as Salmonella which could produce similar colonies on CHROMagar^®^ plates the use of tellurite is necessary (unpublished data).

The identity of potential STEC isolates should be assessed by serotyping and Shiga toxins detection methods. Cell toxicity assay using Vero and HeLa cell lines for Shiga toxin in stool samples or broth enrichment is a very sensitive method since these cell lines have high concentrations of globotriaosylceramides Gb3 and Gb4 which are the receptors for Shiga toxin. Neutralization tests using antibodies against Stx-1 and Stx-2 confirms the results obtained, but this test despite high sensitivity is not routinely used due to its high cost, labor intensity and the expertise required. PCR however offers a fast and reliable method for detection of STEC which similar to immunoassay tests can be used directly with stool samples as well as isolated colonies and depending on the primers used can distinguish between *stx*1 and *stx*2 and detect *eae* and enterohemolysin (*hly*) genes. Use of PCR on DNA extracted from whole stool however, is not recommended because of the low sensitivity (88).

STECs are the only zoonotic *E. coli* pathotype and more than 380 different OH serotypes have now been isolated from humans with gastrointestinal disease and many of these as well as others have been recovered from animals. However, majority of human disease appear to be caused by a limited number of serotypes with frequency varying depending on the location and the year (69, 71–72, 88). Serogroups O26, O45, O91, O103, O111, O113, O121, O145 are listed as the most commonly encountered non-O157 STEC-associated O antigens (69, 88).

#### Epidemiology

Enterohaemorrhagic *E. coli* have been associated with several large outbreaks in US, Canada, Europe and Japan (70, 77), but apart from some sporadic reports on isolating this organism that have not been confirmed by any reference laboratory, no outbreaks or epidemics has been reported from Iran. Detection of this pathotype has been reported in various studies using different screening methods. Neutralization of Stx-1 and Stx-2 was used for identification of STEC strains in various provinces of Iran and showed a varying rate of detection for this pathotype in different locations but O157:H7 serotype was not isolated in any of these provinces (31, 89–90). PCR-detection of STEC isolates have been reported in various studies dealing with diarrhea in Iran with varying rates in different years and study groups (31–34, 67–68, 91–92). Isolation of serologically confirmed O157:H7 was published in 2008, in a study conducted in Zahedan using sorbitol fermentation as primary isolation criteria and serotyping for confirmation. Of the 4 strains that gave positive reaction with antisera against O157, two were identified as O157:H7 (93). However, detection of this bacteria requires an array of different tests and cultures, a combination of molecular and classic methods. Therefore any identification relying on a single method should be considered with caution. Moreover, serotyping for O and H determination especially interpretation of H serology results requires expertise and trained personnel and in view of the fact that so far no epidemics or large outbreaks for this organism have occurred, isolation reports should be assessed more critically.

### Enteroinvasive *E. coli* (EIEC)

#### History

Bacillary dysentery as opposed to dysentery caused by amoeba was described in 1887 and *Bacillus dysenteriae* as the causal agent was described in 1898 by Shiga during an epidemic of 89,400 cases (94). The medical importance of *Shigella* strains led to their separation from *E. coli* and the newly formed genus with its 4 species could be differentiated from *E. coli* on the basis of physiological and biochemical characteristics. However, the discovery of strains which could cause dysentery and were intermediate between *Shigella* and *E. coli* in biochemical characteristic in 1944 caused the separation of the two genera to be questioned (95). The ability of these strains which by now were called enteroinvasive *E. coli* (EIEC) to cause diarrhea was demonstrated in volunteer studies in 1971 (16). It has been shown that EIEC strains and *Shigella* species are biochemically, genetically, and pathogenetically very closely related so much so that it has been proposed that they should be classified as one species in genus *Escherichia* (96–97).

#### Pathogenesis

Acquisition of the invasive plasmid (pINV) encoding the ability to invade host tissues (98–100) is probably the single most important event that has probably given rise to the evolution of both *Shigella* and EIEC from non-pathogenic *E. coli*. Nearly one third of this large single copy plasmid encodes IS elements and contains a 30 kb region enabling the bacteria to invade intestinal epithelial cells (101). Many components of type three secretion system (T3SS) such as translocators, transcriptional activators, some effectors and chaperones are coded by this region with the expression of the Inv-encoded genes being regulated globally by VirB and MxiE (45). In addition to the genes of pINV many chromosomal genes which are not specific to *Shigella* spp. and are carried on the chromosome are required for pathogenesis (101).

Colonic mucosa is the infection site of *Shigella* and EIEC where invasion of M cells, macrophages and epithelial cells occur resulting in a watery diarrhea, which in severe cases may be followed by the onset of scanty dysenteric stools containing blood and mucus (102). EIEC strains may also produce a 63 kDa toxin designated Sen which contributes to the enterotoxic activity detected in the strains carrying the gene (103).

#### Detection

There are very few biochemical characteristics that differentiate *Shigella* and EIEC from each other and the two most convenient are mucate and acetate tests. EIEC may be positive for either or both, whereas with rare exceptions *Shigella* strains are negative for both (97). Salicin fermentation and esculin hydrolysis have also been used to differentiate the two groups (95).

The serotypes associated with EIEC include O28ac, O29, O112ac, O121, O124, O135, O136, O143, O144, O152, O159, O164, O167, and O173 of which O112ac, O124, and O152 are identical to O antigens present in *Shigella* species making identification on the basis of serotyping alone inadequate (97).

The ability to cause keratoconjunctivitis in guinea pig eyes and to form plaques in HeLa cell monolayers were the standard methods of identification for EIEC isolates. However, molecular methods have replaced these phenotypic assays (18) including amplification of a multicopy gene (4-10 copies) called *ipa*H with copies located on both plasmid and chromosome (104–106). This assay distinguishes EIEC and *Shigella* from other diarrheal pathogens but efforts have been made to develop molecular methods to discriminate between the two microorganisms resulting in development of conventional, multiplex as well as real-time PCR methods for this purpose (107–109).

#### Epidemiology

No epidemics and no recent reports of outbreaks caused by EIEC is found in the literature, although some references to older works dealing with outbreaks can be found and due to close similarity between the two organism misidentification is very probable especially in sporadic cases (16).

A mPCR assay targeting *ipa*H was used recently to differentiate EIEC strains from other *E.coli* categories in cases of childhood diarrhea in Tehran (91) showing a 13% isolation rate for *E.coli* of which 19.4% gave positive results with *ipa*H primers.

### Enteroaggregative *E. coli* (EAEC)

#### History

This pathotypes is the most recently identified diarrheagenic *E coli* and is the second most common cause of travelers’ diarrhea after ETEC in both developed and developing countries. EAEC are commonly being recognized as a cause of endemic and epidemic diarrhea worldwide and recently, has been shown to cause acute diarrheal illness in newborns and children in industrialized countries. This organism has also been associated with persistent diarrhea. Diarrhea caused by EAEC is often watery, but it can be accompanied by mucus or blood (43, 110–112).

The discovery of EAEC as well as diffusely adherent *E. coli* (DAEC) stemmed from the studies showing that EPEC adhere to HEp-2 cells in a distinctive pattern (15). Examination of a collection of diarrheal *E. coli* strains that were not of EPEC serogroups showed that many of these strains also adhered to HEp-2 cells and the phenotype was different from that of EPEC (19, 113). This pattern of adherence, which had been called “diffuse” was subsequently subdivided into aggregative and true diffuse adherence (114). *E. coli* showing aggregative adherence (AA) are autoagglutinating, but their hallmark is aggregative adhesion, which involves the formation of a stacked-brick pattern on HEp-2 cells.

In a study in Iran Bouzari *et al*. (115) reported that 32% of diarrheagenic *E. coli* isolated from infants and children which did not belong to any known *E.coli* pathotypes formed AA pattern on HeLa cells and showed a significant prevalence in children with diarrhea compared to controls.

#### Pathogenesis

Lack of suitable animal models and the heterogeneity of virulence factors caused the paucity of details regarding the EAEC transmission, pathogenicity and epidemiology. However, colonization of intestinal mucosa, mucoid biofilm formation and elaboration of various enterotoxins, cytotoxins and mucosal inflammation are considered the major features of EAEC pathogenesis (43, 110–112, 115–116).

Colonization of intestinal mucosa by the EAEC occurs via aggregative adherence fimbriae (AAF) encoded by a 55-65 MDa plasmid named pAA. The first one of which, aggregative adherence fimbriae I (AAF/I), was cloned and characterized from EAEC prototype strain 17-2 (112, 118). A probe derived from this adhesin did not recognize O42, the second EAEC prototype and subsequently a new fimbria was characterized in this strain called AAF/II (119). Although two other adherence factors (AAF/III and AAF/IV) as well as a non-fimbrial adhesin have been described but some strains are encountered that do not contain any of these known fimbriae despite showing AA phenotype which is indicative of the as yet uncharacterized adhesins. (120–122), Similar to ETEC strains adhesion of EAEC to intestinal tissue is mediated by antigenically heterogenous adhesins and multiple carriage of AAFs by an EAEC strain has been rare (123–124). A transcriptional activator known as “AggR,” encoded by pAAs, regulates the biogenesis of AAFs (125) and is the major EAEC virulence regulator controlling diverse virulence genes encoded by pAAs as well as by chromosomes (110, 112). Adherence of EAEC to the mucosa is characterized by the formation of a thick, aggregating mucus layer inside which they survive and this biofilm production has been attributed to the activity of *fis* and *yafK* genes (117, 126). However, a secreted 10 kDa protein encoded by pAA and called antiaggregation protein (Aap) or dispersin, facilitates the movement of bacteria across the surface of the cells for subsequent aggregation and adherence (45, 127). Dispersin is highly immunogenic and is translocated via an ATP binding cassette (ABC) transporter complex (the Aat apparatus) (128). Both these genes have been used for identification and classification of EAEC isolates, but it has been noted that dispersin gene (*aap*) can be detected in DAEC as well as nonpathogenic *E.coli* (129).

#### Detection

The ability of EAEC to form biofilm has been utilized in an assay which has been suggested as a screening test in both clinical and epidemiological studies (16, 110, 130). Formation of biofilm however, was shown to be method dependent and strongly influenced by culture media, leading to the conclusion that considering the experimental variables the results need to be interpreted cautiously (131)

The aggregating nature of this pathovar has made serotyping in many cases impractical and the fraction that can be serotyped belong to a wide range of O:H types, making serotyping of little use in EAEC diagnosis (111).

Bacterial adhesion is followed by the secretion of various toxins of which the plasmid-encoded toxin (Pet), a serine protease causing cytoskeletal rearrangements and EAST1, an activator of guanylate cyclase, are regulated by AggR.

Within EAEC group, different pathogenicity islands have been identified including *she* pathogenicity island of *Shigella*, containing enterotoxin and mucinase genes, and *Yersinia* high-pathogenicity island, containing the yersinibactin siderophore gene (111, 132–133). None of these genes however is present in all the EAEC strains and many of them are not specific for this *E. coli* category which makes developing an alternative method to HEp-2 cell adherence assay difficult. In 1990 a diagnostic probe obtained from the aggregative plasmid of strain 17-2 was reported by Baudry *et al*. (134). The cryptic 1-kb probe known as “CVD432” or aggreagative probe (AA) which was later shown to correspond to the site of Aat transporter complex (112) performed variably in different locations (16). This probe was used by Bouzari *et al*. (135) on a collection of 98 HeLa cell assay-confirmed EAEC isolates of which only 46 (46.9%) reacted with the probe. A PCR method using primers based on the probe sequence was developed by Schmidt *et al*. (136) and its sensitivity and specificity was reported as similar to the AA probe (16). This method has been used by several authors in Iran for detection of EAEC in patients with diarrhea (33–34, 67–68, 137) but only one report used PCR in combination with HeLa cell adherence (123). This group used CVD432 PCR for the preliminary screening of the isolates which were further analyzed for adherence to HeLa cells and found that the PCR method showed 100% sensitivity and 98.4% specificity.

Heterogeneity among EAEC strains in their carriage of putative virulence factors have been well established (16, 43, 45, 110–112, 123–124), but in view of the pivotal role played by *aggR* in regulating a large number of virulence factors and its location on pAA, strains positive for this gene are called “typical EAEC” and strains lacking pAA, but showing the characteristic stacked-bricks phenotype in HEp-2 cell adherence assay are considered “atypical EAEC” (112, 124). Typical EAEC has been associated with diarrhea, but in an extensive genomic analysis of EAEC strains isolated from a case-control study conducted in Mali with children under the age of 5 suffering from moderate to severe diarrhea presence of *aggR* regulon genes was not correlated with diarrhea (138).

The importance of EAEC in diarrheal diseases in various epidemiological and clinical settings and the unusual degree of heterogeneity among EAEC isolates in carrying various putative virulence factors has been well documented. However, data pertaining to the role of individual factors and their contribution in conferring distinct clinical outcomes are required for a true assessment of EAEC as a human pathogen.

### Diffusely adherent *E. coli* (DAEC)

DAEC is a heterogenous group that generates a diffuse adherence pattern on HeLa and HEp-2 cells and has been associated with the watery diarrhea that can become persistent in young children in both developing and developed countries as well as recurring urinary tract infections (43, 139). It has been shown that the relative risk of diarrhea associated with DAEC increases with age of children from 18 months to 5 years. The intestinal carriage of these strains has also been reported to be widespread in older children and adults. The consequences of this persistence are unknown, but several observations have suggested a potential role in the development of chronic inflammatory intestinal disease (139).

Two types of adhesins mediating the DA pattern have so far been described dividing the DAEC strains into AIDA-I-dependent group and those that their adhesins is encoded by a family of related operons, which include both fimbrial and afimbrial adhesins. These groups of proteins are collectively designated Afa-Dr adhesins (43, 140). The first afimbrial adhesin (*afa*) operon belonging to this group was characterized and sequenced in 1984 (141), and subsequently another operon in this family as well as the adhesins receptor were described (142–143). AIDA-I is a 100 kDa outer membrane protein which is associated with DA phenotype and was described by Benz et al. (144) who also showed that this adhesin was not commonly encountered among DEAC isolates (16, 145). The *afa/dr/daa* operons are genes that arise and are expressed in a variety of genetic backgrounds (139) and the pathogenesis of DAEC seems to be predominantly mediated through Afa/Dr adhesin interactions with host cells. In addition a secreted autotransporter toxin (Sat) has also been implicated in pathogenesis, but nevertheless, the implication of Afa/Dr DAEC strains in diarrhea remains controversial. Phenotypic detection of DEAC is based on the mannose-resistant diffuse adhesion of these strains to cultured epithelial HEp-2 or HeLa cells (16, 113–114). The adhesion assay however, is not specific for Afa/Dr DAEC detection, since other pathogenic *E. coli* including EPEC strains may show this pattern of adhesion (21, 140). Other phenotypic assays have also been developed, but none has proved convenient and universal to be used for identification of all Afa/Dr DAEC isolates (140). Colony hybridization using various probes have also been developed and used in epidemiological studies (146–148), but this technique is laborious and time consuming and not suitable for use on individual strains. Design of PCR methods that allow identification of all known Afa/Dr adhesins has been achieved (149, 150), but even with this simpler and faster method no report of Afa/Dr DAEC isolation in Iran has been published.

#### Concluding remarks

A wealth of data concerning the virulence mechanisms of diarrheagenic *E. coli* has been accumulated over the years even though these complicated phenomena are not yet fully understood. This versatile organism affects a wide range of eukaryotic cell processes via an array of diverse genetic elements enabling each pathotype to colonize, multiply, and disseminate and understanding each pathogenic step at molecular level may help in devising effective measures for intervention in infection. However, the contribution from Iran to the global knowledge regarding these pathotypes seems very limited which could be due either to the insignificant role these pathogens play in the public health in this country or the paucity of well designed systematic epidemiological studies and absence of a surveillance system for diarrheagenic *E.coli*. Therefore, to obtain a clear picture of the importance of different diarrheagenic *E. coli* pathotypes in this country and also in order to be able to detect outbreaks quickly and intervene appropriately presence of a network of public health laboratories with trained personnel and validated materials and standardized techniques seems necessary.
